# Supplementary material for: Crawling and Gliding: A Computational Model for Shape-Driven Cell Migration
Source: PLoS Comput Biol. 2015 Oct 21;11(10):e1004280. doi: 10.1371/journal.pcbi.1004280 (PMC4619082; doi:10.1371/journal.pcbi.1004280)
Supplement: S1 Code — (ZIP) [file pcbi.1004280.s012.zip › release/tst/doc/html/warning_8h_source.html]

Tissue Simulation Toolkit: warning.h Source File


Tissue Simulation Toolkit
 0.1.4.1


- Main Page
- Namespaces
- Classes
- Files

- File List
- File Members

warning.h

Go to the documentation of this file.

1 /\*

2

3 Copyright 1996-2006 Roeland Merks

4

5 This file is part of Tissue Simulation Toolkit.

6

7 Tissue Simulation Toolkit is free software; you can redistribute

8 it and/or modify it under the terms of the GNU General Public

9 License as published by the Free Software Foundation; either

10 version 2 of the License, or (at your option) any later version.

11

12 Tissue Simulation Toolkit is distributed in the hope that it will

13 be useful, but WITHOUT ANY WARRANTY; without even the implied

14 warranty of MERCHANTABILITY or FITNESS FOR A PARTICULAR PURPOSE.

15 See the GNU General Public License for more details.

16

17 You should have received a copy of the GNU General Public License

18 along with Tissue Simulation Toolkit; if not, write to the Free

19 Software Foundation, Inc., 51 Franklin St, Fifth Floor, Boston, MA

20 02110-1301 USA

21

22 \*/

23 #ifndef WARNING\_H\_

24 #define WARNING\_H\_

25

26 #define MEMORYCHECK(x) if ((x)==NULL) { fprintf(stderr, "Out of Memory error in "#x" \n"); exit(0); }

27

28 #define UNIDENTIFIED 2353996

29 //static int last\_value=UNIDENTIFIED;

30 /\*#define WATCH(x) if (last\_value==UNIDENTIFIED) { last\_value=x;

31 } else { if (x!=last\_value) { fprintf(stderr,"WATCH value changed. Suspending execution. \n Interrupt within debugger to examine position in program.\n");

32  last\_value=x;

33  while(1);

34  } else {

35  last\_value=x;

36  }

37  }\*/

38

39

40 /\* These functions were a gift from Josh Barnes \*/

41 /\* I changed the name "eprintf" to "warning" \*/

42

43 #ifdef \_\_cplusplus

44 extern "C" {

45 #endif

46

47 void error(char \*, ...);

48 void warning(char \*, ...);

49

50 #ifdef \_\_cplusplus

51 }

52 #endif

53

54 #endif

error

void error(char \*,...)

**Definition:** warning.cpp:62

warning

void warning(char \*,...)

**Definition:** warning.cpp:80


---

Generated on Thu Aug 14 2014 22:04:01 for Tissue Simulation Toolkit by  

 1.8.6
